# Cultural adaptation and validation of the *Strategies to end seclusion restraint* module of the QualityRights ToolKit*

**DOI:** 10.1590/1518-8345.5638.3520

**Published:** 2022-04-20

**Authors:** Ana Beatriz Rizzo Zanardo, Carla Aparecida Arena Ventura

**Affiliations:** 1 Universidade de São Paulo, Escola de Enfermagem de Ribeirão Preto, Centro Colaborador da OPAS/OMS para o Desenvolvimento da Pesquisa em Enfermagem, Ribeirão Preto, SP, Brasil.; 2 Bolsista da Coordenação de Aperfeiçoamento de Pessoal de Nível Superior (CAPES), Brasil.

**Keywords:** Mental Health, Mental Disorders, Human Rights, Physical Restraint, Validation Study, Inservice Training, Saúde Mental, Transtornos Mentais, Direitos Humanos, Restrição Física, Estudo de Validação, Capacitação em Serviço, Salud Mental, Trastornos Mentales, Derechos Humanos, Restricción Física, Estudio de Validación, Capacitación en Servicio

## Abstract

**Objective:**

to adapt to the Brazilian culture and validate the module *“Strategies to end seclusion restraint”* of the *QualityRights* toolkit of the World Health Organization on mental disorders to train health professionals in Brazil.

**Method:**

it is a methodological study divided into three stages. The modules were translated from the original language (English) to the target language (Brazilian Portuguese) in the first stage. In the second, the translation was assessed by a committee of judges with seven experts. In the third stage, the assessment was conducted by mental health professionals (nurses, psychologists, and lawyers), in which seven mental health professionals participated. They assessed the material using the *Suitability Assessment of Materials* instrument.

**Results:**

in the assessment conducted by the experts (n=7), 8 items assessed obtained 100% approval and the other 6 items obtained lower approvals, with a total approval of 92%. Regarding the assessment by health professionals (n=7), 2 items had a maximum approval of 100% and the other had the approval of 86% each, with a total approval of 88%.

**Conclusion:**

based on the results, it is considered that this module is adapted to the Brazilian culture and can be used to train Brazilian health professionals.

## Resumo

**Objetivo:**

adaptar para a cultura brasileira e validar o módulo *“Strategies to end seclusion restraint”* do *toolkit QualityRights* da Organização Mundial da Saúde sobre transtornos mentais para a capacitação de profissionais de saúde no Brasil.

**Método:**

trata-se de estudo metodológico, dividido em três etapas. Na primeira, foi realizada tradução dos módulos da língua original (inglês) para a língua-alvo (português brasileiro). Na segunda, foi realizada a avaliação da tradução por um comitê de juízes composto de sete especialistas. Na terceira etapa, foi realizada a avaliação por profissionais de saúde mental (enfermeiros, psicólogos e advogados), da qual participaram sete profissionais de saúde mental. Eles avaliaram o material por meio do instrumento *Suitability Assessment of Materials*.

**Resultados:**

na avaliação realizada pelos especialistas (n=7), 8 itens avaliados obtiveram 100% de aprovação e os outros 6 itens aprovações menores, obtendo-se uma aprovação total de 92%. Quanto à avaliação realizada pelos profissionais de saúde (n=7), 2 itens tiveram aprovação máxima de 100% e os demais tiveram aprovação de 86% cada um, obtendo-se aprovação total de 88%.

**Conclusão:**

com base nos resultados, considera-se que este módulo está adaptado para a cultura brasileira, podendo ser utilizado para a capacitação de profissionais de saúde brasileiros.

## Resumen

**Objetivo:**

adaptar a la cultura brasileña y validar el módulo *“Strategies to end seclusion restraint* del *‘toolkit QualityRights’”* de la Organización Mundial de la Salud sobre trastornos mentales para la capacitación de profesionales de la salud en Brasil.

**Método:**

se trata de un estudio metodológico, dividido en tres etapas. En la primera, fue realizada la traducción de los módulos de la lengua original (inglés) a la lengua objetivo (portugués brasileño). En la segunda, fue realizada la evaluación de la traducción por un comité de jueces compuesto de siete especialistas. En la tercera etapa, fue realizada la evaluación por profesionales de la salud mental (enfermeros, psicólogos y abogados), de la cual participaron siete profesionales de la salud mental. Ellos evaluaron el material por medio del instrumento *Suitability Assessment of Materials*.

**Resultados:**

en la evaluación realizada por los especialistas (n=7), 8 ítems evaluados obtuvieron 100% de aprobación y los otros 6 ítems aprobaciones menores, obteniéndose una aprobación total del 92%. En cuanto a la evaluación realizada por los profesionales de la salud (n=7), 2 ítems tuvieron aprobación máxima del 100% y los demás tuvieron aprobación del 86% cada uno, obteniéndose aprobación total del 88%.

**Conclusión:**

con base en los resultados, se considera que este módulo está adaptado a la cultura brasileña, pudiendo ser utilizado para la capacitación de profesionales de la salud brasileños.

**Highlights**

(1) The paper is the first study to validate one of the modules of the *QualityRights* Initiative.

(2) It aims to promote the human rights of people with mental disorders.

(3) It promotes alternatives to the non-use of coercive practices.

(4) It was validated with the target audience, ensuring it was understandable for everyone.

## Introduction

Currently, despite considerable advances in research on how to prevent and treat people with mental disorders, respecting their dignity and diversity, besides promoting mental health, the translation of these assumptions into real-world effects has been slow. The global burden of mental disorders has increased in all countries, especially in the context of major demographic, environmental, and sociopolitical transitions. Additionally, human rights violations and abuses of this population persist, with many people locked away in mental health services and prisons or living on the streets without any political and legal protection. Still, the quality of mental health services is worse than that of other health services in general, as government investment and mental health care remain small^1^.

Among the risk factors that can lead to the development of a mental disorder, the following are emphasized: physical, sexual, and emotional abuse, neglect, poverty, loss of a parent, domestic violence, serious physical illness, exposure to parental mental illness, and misuse of substances^2^. People living in low- and middle-income countries are exposed to many factors that make them vulnerable to the development of mental disorders^3^.

Concerning the treatment possibilities, different principles for mental health care advocate that each institution should provide a therapeutic environment that supports a culture of recovery, individual training, and responsibility, in which the patient has an active voice in determining their treatment options^4^. However, a study conducted in Norway concluded that 13 people per 100,000 inhabitants *per* year (1.7% of hospitalized patients) were submitted to restraint in the wards in the eight-year period from 2004 to 2011 in the country^5^. 

Physical restraint involves direct physical contact between people, a procedure in which force is applied against resistance, either to restrict movement or mobility or to get rid of the harmful behavior presented by an individual. Chemical restraint includes the use of medicines, and mechanical restraint includes the use of equipment^4^. Physical restraints cause a series of severe clinical and ethical problems, violating the patient’s autonomy, the principles of justice, beneficence, and non-maleficence, since restraints can cause physical injuries, including skin lesions, damage to the nervous system, lung disease, deep vein thrombosis, or even death, in addition to causing trauma to health professionals, therefore, such practice should be considered illegal^6^.

Thus, it is relevant to ensure that people with mental disorders enjoy different means of access to the exercise of their rights to deal with their growing vulnerability and social exclusion^7^. The training of mental health services and different actors, such as health professionals, people with mental disorders, their families, and the population in general, is an urgent need that has not been able to attract sufficient attention and funding^8^.

Thus, the World Health Organization (WHO) developed the *QualityRights* initiative, with an initial emphasis on promoting sustainable changes in attitudes and practices in mental health and ensuring respect for the human rights of people with mental health problems and psychosocial disabilities. An important goal of the WHO *QualityRights* initiative is to provide practical solutions to promote human rights in all mental and social health systems and, in particular, support countries in enforcing the rights of the Convention. *QualityRights* resources are designed to train people in mental health, focusing on respect for human rights and recovery, reaching a wide range of stakeholders^9^.

The *QualityRights* project is the result of the efforts of an international working group, divided into two parts, the first consists of the formatting and dissemination of a “toolkit” to support States in assessing and improving quality and respect for human rights in national, regional, and local mental health and social assistance services^10^; the second part of the *QualityRights* Initiative refers to training and guidance materials, which can be used to train mental health professionals, people with psychosocial, intellectual, and cognitive disabilities, people who use mental health services, families, caregivers and other supporters such as non-governmental organizations, organizations of people with disabilities and others, on how to implement a human rights and recovery approach in the area of mental health and in line with the Convention on the Rights of Persons with Disabilities (CRPD) and other international human rights standards^11^.

These resources include five basic training modules addressing human rights, mental health, disability, capacity, recovery, and the right to freedom from coercion, violence, and abuse. There are also three specialized training modules addressing recovery practices, strategies to end seclusion and restraint, and support in decision making and early planning. These modules are designed to be taught in workshops conducted by multidisciplinary teams, including people experienced with mental disorders. They are addressed to all people involved in mental health services, from service users and family members to physicians and managers. The modules are flexible and can be adjusted to meeting the participants’ needs^12^.

A study that showed the application of the *QualityRights* training program in Iceland reports that the results revealed changes in attitude among the participants after completing the program. High levels of attitude change are demonstrated in more than three quarters of the statements from the pre-test to the post-test of the training. The use of coercive practices such as chemical restraint also presented changes in attitude^13^. In this perspective, to improve the quality and safety of care in psychiatric hospitals and reduce restrictive measures, it is recommended to use the personnel training led by the WHO^14^.

This study has the following guiding question: *“Will the validation and cultural adaptation of* QualityRights *modules be understandable aiming at training health professionals in Brazil?”.* It aims, therefore, from its validation, to contribute to the care provided in health services throughout the national territory, promoting knowledge and demonstrating means to ensure the exercise of human rights of people with mental disorders for all actors involved in care, including people with mental disorders themselves; thus, it highlights strategies to reduce the use of coercive measures such as seclusion and restraint, improving the quality of life and care provided to people with mental disorders. 

Therefore, the validated modules will be important allies in the training of health professionals in Brazil, especially in the category of nursing professionals, who constitute the largest class of health professionals in the country, playing a unique, crucial and close role to people with mental disorders, because the modules allow professionals to improve the care offered, based on the guarantee of human rights and the principles of ethics, considering the patient as the main actor of their life and recovery.

The research aimed to adapt to the Brazilian culture and validate the module “Strategies to end seclusion restraint” of the QualityRights toolkit of the World Health Organization on mental disorders to train health professionals in Brazil. 

## Method

### Type of study

This is a methodological study focused on developing, assessing and improving an instrument or strategy^15^, organized according to the *Revised Standards for Quality Improvement Reporting Excellence* (SQUIRE 2.0) guidelines of the *Equator* Network. This type of study aims to elaborate, assess, and validate the technologies created to ensure their reliability for use in educational and care environments^16^. The methodological proposal for validating the modules was adapted for this study based on the models of Beaton^17^ and Pasquali^18^. The stages completed are described below:

1. Translation^17^;
2. Analysis of the Judges^18^;
3. Pre-test^18^.

### Study stages

*1* ^st^ *Stage: Translation:* the modules were translated from the original language (English) into the target language (Brazilian Portuguese) by two independent translators, native to the target language and with different occupations. This technique makes it possible to detect divergent errors and interpretations. This process aims to preserve the meaning of each item between the two languages and maintain the integrity of the measuring instrument^17^. After analyzing the two versions of translations, there was a consensus, reaching a single version, called the *final version1*.

*2* ^nd^ *Stage: Committee of judges:* a total of *ten* experts on the themes of this study were invited; however, two responses were not obtained and one of them refused to participate. Thus, seven experts agreed to participate, receiving the final version of the module’s translation to read, analyze, and assess the material through the *Suitability Assessment of Materials* (SAM) scale (adapted for this study). For this type of validation, six to ten experts are recommended^19^. After this stage, the analysis of the data sent by the experts and the adjustments in the module were conducted, resulting in the *final version2*.

*3* ^rd^ *Stage: Pre-test: seven* professionals from a Psychosocial Care Center in the countryside of the state of São Paulo were invited; however, only four professionals analyzed the material and sent it back to the researchers. Mental health professionals from other services were also invited, using the convenience sampling technique^15^ ^-^ ^16^. Nine more health professionals from other mental health services were invited. Among these, four did not respond to the email if they would accept to participate in the research; two health professionals agreed to participate in the research but did not send the analyzed material back to the researchers and *three* agreed to participate in the research and sent back the analyzed material.

In total, seven health professionals participated in this stage and they received the *final version2* of the translation of the module to read, analyze, and assess the material through the *Suitability Assessment of Materials* (SAM) scale (adapted for this study). For this type of validation, six to ten participants are recommended^19^.

After data analysis, the suggestions of the professionals were grouped, analyzed, and accepted. After the modifications suggested by them were made, a new version of the module was obtained. It was the last version, that is, the *final version3*.

**Figure 1:**
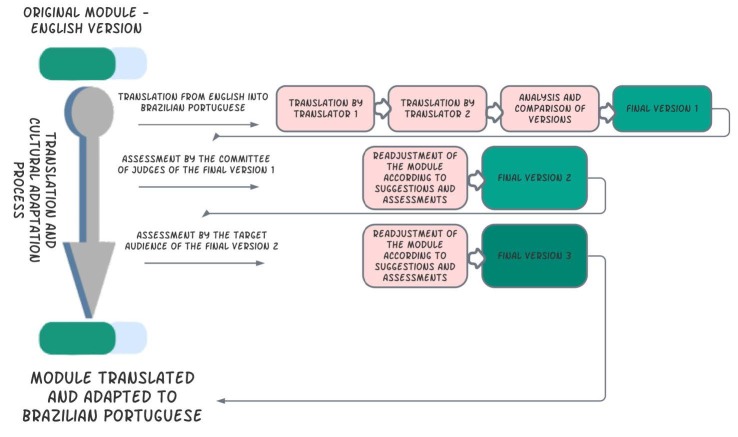
Stages of the translation, cultural adaptation, and validation process. Ribeirão Preto, SP, Brazil, 2021

### Instruments used to collect information

To verify the agreement of the participants in relation to the items of the analysis, the adaptation of the *Suitability Assessment of Materials* (SAM) instrument was used, which includes questions to assess the difficulty and convenience of educational materials^20^, adapted and culturally validated^21^.

The American instrument called SAM consists of a list or checklist with six categories (content, understanding of the text, illustration, presentation, motivation and cultural adaptation). The application requires less than 15 minutes. The sum of the points attributed to each instrument item categorizes the material as to the suitability for the target audience^21^.

According to how it was adapted for this study, the instrument presented the following subcategories: In the item Content: Is the objective evident, facilitating the understanding of the material? The content addresses information related to mental disorders and human rights; Is the material proposal suitable to the objectives? In the item Language: Is the information passed on within a clear context? Is the reading level suitable for the reader’s understanding? Does the vocabulary use common words? Is learning facilitated by topics? In the Graphic illustrations item: Are the illustrations relevant? In the item Presentation: Is the material organization suitable? Are the font size and font suitable? In the item Stimulation/Motivation: Are the desired behavior patterns well modeled or demonstrated? Is there motivation to change behavior? In the item Cultural Suitability: Is the material culturally suitable to the logic, language, and experience of the target audience? Does it present culturally suitable examples?

To better understand the participants, the Likert scale was used, a psychometric scale that intends to record the level of agreement or disagreement of a given information^22^ ^-^ ^23^. 

### Data processing and analysis

The professional information about the experts and health professionals and their sociodemographic data were coded and typed in spreadsheets of the Excel application. Descriptive analysis was performed with the calculation of frequencies and percentages through the IBM SPSS *Statistics* v.25 software.

To analyze the data of the assessment performed by the SAM instrument, the mean agreement between participants was calculated^24^ through the content validity index (CVI), which is a measure calculated based on the representativeness of positive responses through various methods. First, it is recommended to calculate the CVI for each item, counting the number of experts or professionals who classified the item as three or four and dividing this number by the total number of experts. This results in the proportion of experts who considered the item as valid content^25^.

Thus, the CVI measures the proportion or percentage of judges who agree on certain aspects of the instrument and its items. Items marked with “Agree” or “Strongly Agree” are considered representative, obtaining an index score of 1.00 and the item that has all assessments equal to 1.00 will have 100% agreement^26^.

To perform this calculation, the responses of the items were regrouped and the values that corresponded to 0 and 1 on the Likert scale (strongly disagree and disagree) started to correspond to (-1); when the value was 2 (neutral/indifferent), it became (0) and when the values were 3 and 4, it became (+1). Thus, the response of each judge could vary from -1 to +1, and the closer to +1, the greater the agreement that the item was pertinent. Based on these responses, it was possible to calculate the means of agreement of the committee of judges, calculating the percentage of responses corresponding to the value +1^24^. For the item to be approved, an agreement of at least 80% among professionals is required^18^.

The assessment of the instrument in its entirety does not require consensus in the literature. Besides, there are three ways in which the mean of the proportions of the items considered relevant by the judges; the mean of the values of the items calculated separately, that is, all CVIs calculated separately are added and divided by the number of items considered in the assessment. The last way would be to divide the “total number of items considered relevant by the judges by the total number of items”. In the case of six or more, a rate of not less than 0.78 is recommended^19^. Thus, it was decided to divide the total number of items considered relevant (with response 1 - agree or strongly agree) by the total number of items answered in the instrument.

The SAM instrument allows participants to express opinions and suggestions on each item. Thus, the experts’ suggestions were grouped, analyzed and reasonable modifications were made. 

### Ethical aspects

This study was approved by the Research Ethics Committee of the University of São Paulo at Ribeirão Preto College of Nursing (CAAE: 21828819.4.0000.5393 and Opinion Number: 3,743,964). The Free and Informed Consent Form was given to the committee of judges and the health professionals who participated in the study.

## Results

Regarding the Committee of Judges, of the seven participating experts, all (100%) were female. The age of each expert varied, with a mean of 42.3 years. Regarding the professions, one expert (14.3%) was a lawyer, three (42.9%) were nurses, one (14.3%) was a nurse and university professor and two (28.6%) were psychologists. The time since graduation also ranged from seven years to 31 years, with a mean of 19.1 years.

Regarding the degree, four (57.1%) had a doctoral degree, two (28.6%) had a master’s degree and one (14.3%) had a postdoctoral degree.

The seven experts (100%) participated in a training course in the last five years, with a mean of 9.3 participations; all (100%) published scientific articles in the last five years with a mean of 6.6 publications. Besides, the seven experts (100%) stated that they presented works in the last five years, with a mean of 21.1 presentations per expert.

The tables in sequence summarize the participants’ information.

**Table 1:** Participants in the Committee of Judges stage (n=7) according to sociodemographic characteristics. Ribeirão Preto, SP, Brazil, 2021

Characteristics of Study Participants (Committee of Judges)	% of respondents (N=7)		
**Age (years old)**			
31	14.3	Mean	42.3
33	14.3	Median	43.0
36	14.3	Standard Deviation	9.2
43	14.3	Minimum	31
47	14.3	Maximum	53
53	28.6		
**Sex**			
Female	100.0		
**Profession**			
Lawyer	14.3		
Nurse	42.9		
Nurse and University Professor	14.3		
Psychologist	28.6		
**Time since graduation (years)**			
7	14.3	Mean	19.1
10	14.3	Median	19.0
13	14.3	Standard deviation	9.5
19	14.3	Minimum	7
25	14.3	Maximum	31
29	14.3		
31	14.3		
**Current working time (years)**			
0.08	14.3	Mean	12.05
0.25	14.3	Median	6
4	14.3	Standard deviation	12.55
6	14.3	Minimum	0.08
18	14.3	Maximum	31
25	14.3		
31	14.3		
**Title/Degree**			
Master’s	28.6		
Doctorate	57.1		
Post-doctorate	14.3		
**Do you work in the area of interest?**			
Yes	100.0		
**What is the area of interest?**			
Teaching	14.3		
Mental health	71.4		
Mental Health/Scholar	14.3		
**Participation in training courses**			
Yes	100.0		
**Amount of participation**			
2	14.3	Mean	9.3
3	14.3	Median	8.0
5	14.3	Standard deviation	6.9
8	14.3	Minimum	2
10	14.3	Maximum	20
17	14.3		
20	14.3		
**Article publication in the last 5 years**			
Yes	100.0		
**Number of articles published**			
2	28.6	Mean	6.6
6	14.3	Median	7.0
7	14.3	Standard deviation	3.8
8	28.6	Minimum	2
13	14.3	Maximum	13
**Presentation of works in the last 5 years**			
Yes	100.0		
**Number of presentations**			
2	14.3	Mean	21.1
3	14.3	Median	8.0
6	14.3	Standard deviation	36.8
8	14.3	Minimum	2
12	14.3	Maximum	104
13	14.3		
104	14.3		

Regarding Health Professionals, six participants (85.7%) were female and one (14.3%) was male. The age of each participant ranged from 25 to 48 years, with a mean age of 35.9 years. Regarding the professions, one professional (14.3%) was a social worker, two (28.6%) were nurses, one (14.3%) was a psychiatric nurse, two (28.6%) were psychologists and one (14.3%) was an occupational therapist. The time that the professionals were working or had already worked in mental health also varied for each expert, between a minimum of six months and a maximum of 24 years and the mean was 9.5 years, as shown in the following table (Table 2).

**Table 2:** Health professionals (n= 7) according to sociodemographic characteristics. Ribeirão Preto, SP, Brazil, 2021

Characteristics of Health Professionals participating in the study	% of respondents (N=7)		
**Age (years old)**			
25	14.3	Mean/Median	35.9/35.0
27	14.3	Standard deviation	8.5
32	14.3	Minimum	25
35	14.3	Maximum	48
41	14.3		
43	14.3		
48	14.3		
**Sex**			
Female	85.7		
Male	14.3		
**Profession**			
Social Worker	14.3		
Nurse	28.6		
Psychiatric Nurse	14.3		
Psychologist Occupational therapist	28.6 14.3		
**Time since graduation (years)**			
2	14.3	Mean/Median	11.3/12.0
4	14.3	Standard deviation	7.5
10	14.3	Minimum	2
12	28.6	Maximum	25
14	14.3		
25	14.3		
**Time working/worked in the mental health (years)**			
0.50	14.3	Mean/Median	9.58/9
0.58	14.3	Standard deviation	8.79
5	14.3	Minimum	0.5
9	14.3	Maximum	24
10	14.3		
18	14.3		
24	14.3		
**Title/Degree**			
Bachelor’s and Teaching Degree	14.3		
Specialization/Residency	42.9		
Doctorate	28.6		
Specialization/Residency and master’s degree	14.3		
**Do you work in the area of interest**			
No answer	14.3		
Yes	85.7		
**What is the area of interest?**			
Research	14.3		
Mental health	71.4		
No answer	14.3		
**Participation in training courses**			
Yes	85.7		
No	14.3		
**Amount of participation**			
0	14.3	Mean/Median	3.9/3.0
1	14.3	Standard deviation	3.6
3	28.6	Minimum	0
4	14.3	Maximum	11
5	14.3		
11	14.3		
**Article published in the last 5 years**			
Yes	42.9		
No	57.1		
**Number of articles published**			
0	57.1	Mean/Median	1.3/0.0
2	28.6	Standard deviation	1.9
5	14.3	Minimum	0
		Maximum	5
**Presentation of works in the last 5 years**			
Yes	42.7		
No	57.1		
**Number of presentations**			
0	57.1	Mean/Median	1.6/0.0
2	14.3	Standard deviation	2.1
4	14.3	Minimum	0
5	14.3	Maximum	5

Regarding the assessment conducted by the experts, of the 14 items assessed, 8 obtained 100% approval (57.14% of the items) and in 2 of them (Q6 and Q8), the lowest approvals (71%) were obtained. The total approval percentage of the module was 92%. 

Regarding the assessment conducted by health professionals, the percentage of approval varied by item and two (Q9 and Q10) - corresponding to 14.2% of the items - had a maximum approval of 100% and the other (corresponding to 85.71% of the items) had the approval of 86% each. The total approval percentage of the module was 88%. 

The results of the analysis of the judges and health professionals can be seen in the following Table 3.

**Table 3:** Analysis of the committee of judges (n=7) and health professionals (n=7) according to the approval. Ribeirão Preto, SP, Brazil, 2021

Judge/Assessment in each question (Q1; Q2...)	J1	J2	J3	J4	J5	J6	J7	CVI*	**Approval percentage *per* item**
**Q1 - Is the objective evident, facilitating the understanding of the material?**	1	1	1	1	1	1	1	1.0	100%
**Q2 - Does the content address information related to mental disorders and human rights?**	1	1	1	1	1	1	1	1.0	100%
**Q3 - Is the material proposal suitable to the objectives?**	1	1	1	1	1	1	1	1.0	100%
**Q4 - Is the information passed on within a clear context?**	1	1	1	1	1	1	1	1.0	100%
**Q5 - Is the reading level suitable for the reader’s understanding?**	1	1	1	1	1	1	0	0.86	86%
**Q6 - Does the vocabulary use common words?**	1	0	1	1	1	1	0	0.71	71%
**Q7 - Is learning facilitated by topics?**	1	1	1	1	1	1	1	1.0	100%
**Q8 - Are the illustrations relevant?**	0	1	1	1	1	-1	1	0.71	71%
**Q9 - Is the material organization suitable?**	1	1	1	1	1	1	1	1.0	100%
**Q10 - Are the font size and font suitable?**	1	1	1	1	1	1	1	1.0	100%
**Q11 - Are the desired behavior patterns well modeled or demonstrated?**	1	1	1	1	1	1	0	0.86	86%
**Q12 - Is there motivation to change behavior?**	1	1	1	1	1	1	1	1.0	100%
**Q13 - Is the material culturally suitable to the logic, language, and experience of the target audience?**	1	1	1	1	1	1	0	0.86	86%
**Q14 - Does it present culturally suitable examples?**	1	0	1	1	1	1	1	0.86	86%
**Total Approval**								0.92	92%
**Health Professionals/Assessment on each question (Q1; Q2...)**	P1	P2	P3	P4	P5	P6	P7	CVI	**Approval percentage *per* item**
**Q1 - Is the objective evident, facilitating the understanding of the material?**	1	1	1	1	1	1	0	0.86	86%
**Q2 - Does the content address information related to mental disorders and human rights?**	1	1	1	1	1	1	0	0.86	86%
**Q3 - Is the material proposal suitable to the objectives?**	1	1	1	1	1	1	0	0.86	86%
**Q4 - Is the information passed on within a clear context?**	1	1	1	1	1	1	0	0.86	86%
**Q5 - Is the reading level suitable for the reader’s understanding?**	1	1	1	1	1	1	0	0.86	86%
**Q6 - Does the vocabulary use common words?**	1	1	1	1	1	1	0	0.86	86%
**Q7 - Is learning facilitated by topics?**	1	1	1	1	1	1	0	0.86	86%
**Q8 - Are the illustrations relevant?**	1	1	1	1	1	1	0	0.86	86%
**Q9 - Is the material organization suitable?**	1	1	1	1	1	1	1	1.0	100%
**Q10 - Are the font size and font suitable?**	1	1	1	1	1	1	1	1.0	100%
**Q11 - Are the desired behavior patterns well modeled or demonstrated?**	1	1	1	1	1	1	0	0.86	86%
**Q12 - Is there motivation to change behavior?**	1	1	1	1	1	1	0	0.86	86%
**Q13 - Is the material culturally suitable to the logic, language, and experience of the target audience?**	1	1	1	1	1	1	0	0.86	86%
**Q14 - Does it present culturally suitable examples?**	1	1	1	1	1	1	0	0.86	86%
**Total Approval**								0.88	88%

*CVI = Content Validity Index

Still, the committee of judges and health professionals made suggestions regarding the material, summarized in the following figure:

**Figure 2:** Suggestions from study participants. Ribeirão Preto, SP, Brazil, 2021

Main suggestions of the judges (J1; J2...) in FV2*	Main suggestions of health professionals (P1; P2...) in FV3^†^
*(...) I suggest reflecting on the vocabulary/language presented, mainly in the initial topics.* **(J7)**	*Continuous reading becomes tiresome, but it becomes interesting and enlightening for items. The translation was performed very well, and reading is possible without reservations.* **(P3)**
*It would be interesting to invest in mental maps to facilitate the understanding of elementary and middle-level professionals.* **(J2)**	*The final graphic assists in understanding the material as a whole.* **(P4)**
*The material does not contain illustrations (photos, images, and figures). However, considering the tables and flowchart that appear as illustrations, we can consider them relevant. Link images can also be considered relevant.* **(J1)**	*I found the reading only a little extensive, tables and summary helped in the synthesis and overview of the set* **(P6)**
*I did not see the need for illustrations of the material, in addition to the QualityRights initiative symbol.* **(J6)**	*I believe that just by reading it becomes difficult, motivating the practical experience with this material would be richer. (...) Very rich material, but the reading becomes tiresome and extensive.* **(P4)**
*It would be interesting to include clearer and more frequent examples in the Brazilian reality.* **(J2)**	*I could attach real Brazilian cases to exemplify the topics and approach culturally.* **(P5)**
*The material content is very rich!!! (...) I believe that the topic of the module that will imply greater implementation challenges will be Topic 4- “Challenging assumptions about seclusion and restraint”, as it involves everyday beliefs and situations of health services related to experiences of violence, anchored in fear and dangerousness and justified by the desire to protect. It may be interesting to encourage, indeed, the exposure of own examples, using exercises with strategies that involve more sensitivity, emotions*. **(J5)**	*The material is excellent, it can be adapted to various target audiences, bringing the discussion of a topic of extreme importance in mental health services in Brazil.* **(P2)**

^*^Final version 2; ^†^Final version 3.

## Discussion

The discussion was divided into two categories: *Understanding the analysis of the Committee of Judges* and *Understanding the analysis of Health Professionals*.

### Understanding the analysis of the Committee of Judges

It is necessary to validate educational materials to create a version that achieves equivalence between the original and translated versions. The translation and validation process requires high methodological rigor that consists of the instrument’s translation and revision by a committee of experts^27^. The validation process with assessing items related to the objective, structure/presentation and relevance is vital so that educational materials do not have mistaken or incomplete information, that can mislead the target population or hinder the understanding of the theme^28^. After translation, assessment by the panel of experts is an important stage to identify terms that the target audience may not understand and, thus, change them^29^.

The Committee of Judges of this study assessed a module of the *QualityRights* Initiative. The items that obtained agreement indexes between the judges greater than or equal to 80% were considered validated^18^ ^,^ ^24^. Although the literature indicates that an agreement index greater than or equal to 70% is sufficient for an item to be considered validated, a higher agreement index was chosen, since there is no knowledge of studies already conducted validating educational modules such as this one^24^.

Some categories did not achieve 100% approval in this process, obtaining less than 80%. To improve the assessments and achieve an excellent acceptability index of the module regarding its cultural adaptation, all changes were based on the judges’ suggestions, besides the assessments conducted. In addition, it was decided not to insert more illustrations due to the suggestions of some judges, for whom the illustrations were unnecessary.

As in other material validation studies, the main suggestions for modifications were concerning the wording of the items, such as the substitution of some words and standardization of others^30^, which allowed the suitability of the terminology used and clarification of the information transmitted, thus ensuring the consistency of the textual and visual resources of the material^31^.

It is important to draw attention to the domains with the lowest proportion of positive responses (71%). This fact can be explained due to the amount of information presented in the material. Nevertheless, this can be minimized by correctly using the material, offering it as a course associated with health education actions, not only reading^32^. It is noteworthy that, despite these lower assessments, the items did not receive negative assessments from any judge; however, adjustments were made in the translations of the words/phrases to enable a more understandable language for the Brazilian population. As in another study, some items achieved lower scores, being corrected, guided by the judges’ recommendations, and the content was reviewed for the scientific basis, the cultural context of the target audience and grammatically reviewed^33^. 

Regarding the validation process, the professional diversity of the committee of judges proved to be a very favorable factor since it grouped different knowledge of different specialties within the theme addressed by the module, resulting in a multidisciplinary and complete work^34^. Thus, the semantic, idiomatic, cultural and conceptual equivalences obtained by the assessment of a committee of experts in relation to the original Brazilian Portuguese version were certified and good content validity indexes assessed by the committee of judges were obtained^35^. All suggestions were accepted or justified and the module received total approval of 92%, being considered culturally adapted and validated for Brazilian culture, according to the face and content validation by the committee of judges.

### Understanding the analysis of health professionals

Besides having correct information and being valid concerning the content, health education materials must be understandable by the target audience. In this perspective, health professionals experienced with mental health conducted the content validation and suggested minor adjustments^28^ ^,^ ^36^.

It is essential to highlight that the planning of this study was built in a systematic way to understand the scenario and facilitate the approach and the type of action for the transformation of practice and, consequently, of reality, seeking to maintain the creative, consistent, and innovative didactic design that the material already had^37^.

The assessment conducted by the team of mental health professionals of this investigation resulted in some items with maximum approval (100%) and others with 86% approval. After the necessary adjustments were made, the target audience (health professionals) positively assessed the material in the previous stage, considering it interesting, interactive, explanatory, and motivating. This assessment showed that the material could be used for Brazilian health professionals, with levels of agreement of positive responses greater than 80%^32^.

The study results approached the validation of another material validation research, in which a positive level of agreement equal to 88.4% was observed in the responses of the target audience. Despite being a good result, the difficulty faced when dealing with such a demanding audience is evident, as was also noticed in this study^38^.

The professionals’ suggestions were analyzed and used to modify the content to improve the material related to the cultural adaptation process. This process contributed to enriching the educational proposal, especially in the items that did not have 100% approval since these assessments and suggestions came from professionals who share the purpose of qualification in mental health^39^. The results indicate the importance of investigating the opinion of the target population of the study on the use of materials so that the final version available is as compatible as possible with their autonomy. This corroborates the population’s active participation in the teaching-learning processes on prevention, recovery, and health rehabilitation^36^. As in another study in the literature, the target audience’s contributions and observations were recorded to ensure the best quality of the educational material for the population^38^.

The material approached, used as a course, has great potential to raise awareness of the target audience and needs to be understandable by anyone, clear in its technical-scientific approach. Thus, participants need to understand and apprehend the guidelines to be transmitted, besides being clear, simple, and direct, making communication effective^31^.

Therefore, the importance of this module for the experience of health professionals is emphasized since the constant and dynamic transformations undergone by modern society impose an increase in qualified professional demands, requiring more and more people with specific knowledge and skills to achieve the expected levels of development^40^. Finally, as in another innovative study, it is noteworthy that this Brazilian version of the module is the first to be made available since other validation versions for this specific context and material were not found in the literature^40^. It is clear, then, the need to develop innovative methods such as this one, capable of promoting health actions^41^.

It is noteworthy that the suggestions were widely discussed until the final version was obtained. The analysis of the conceptual and material equivalence between the original version and the one translated and culturally adapted contributed to consolidating a consensual version of the module into Brazilian Portuguese^42^. Therefore, it is considered that the instrument was able to meet the purposes for which it was developed, being able to guide the construction of knowledge in health in different formats^30^. Although the results show that the content of this module is validated by health professionals and is therefore easy to understand for the target audience, it is considered necessary to develop other studies that prove the effectiveness of transforming the reality of this material^43^. It is noteworthy as a limitation the lack of offer of the module as a course for the target audience, requiring, therefore, more studies to test the effectiveness of its application in practice, also considering the question of how this material will affect the knowledge and practices of health professionals.

It is also reinforced that the study can contribute to the advancement of scientific knowledge in health and nursing, improving the care provided based on respect for the human rights of people with mental disorders.

## Conclusion

The module was translated into Brazilian Portuguese by three professionals from different occupations. It was assessed by a committee of judges, experts in the subject, receiving a total approval of 92%. Most of the suggestions were accepted. Soon after, to certify that the material was understandable to the entire population, especially to the target audience, the content was assessed by mental health professionals, receiving a total approval of 82%. Besides that, almost all suggestions were accepted, and those that were not had justifications.

Based on the results evidenced, this module is considered validated and adapted to the Brazilian culture, according to the face and content and can be used, aiming at the training of Brazilian health professionals. It is crucial that, after this validation, the module is offered through a course for mental health professionals, aiming to analyze its effectiveness. 

Finally, it is emphasized that with the results of this study important measures to reduce coercive practices can be implemented in the daily life of services, improving the quality of care provided and the quality of life of people with mental disorders.
